# The Role of China in the Global Spread of the Current Cholera Pandemic

**DOI:** 10.1371/journal.pgen.1005072

**Published:** 2015-03-13

**Authors:** Xavier Didelot, Bo Pang, Zhemin Zhou, Angela McCann, Peixiang Ni, Dongfang Li, Mark Achtman, Biao Kan

**Affiliations:** 1 Department of Infectious Disease Epidemiology, Imperial College London, London, United Kingdom; 2 State Key Laboratory for Infectious Disease Prevention and Control. National Institute for Communicable Disease Control and Prevention, Chinese Center for Disease Control and Prevention, Changping, Beijing, China; 3 Collaborative Innovation Center for Diagnosis and Treatment of Infectious Diseases, Hangzhou, China; 4 Environmental Research Institute and Department of Microbiology, University College Cork, Cork, Ireland; 5 Warwick Medical School, University of Warwick, Coventry, United Kingdom; 6 Binhai Genomics Institute, BGI-Tianjin, BGI-Shenzhen, Tianjin, China; Universidad de Sevilla, SPAIN

## Abstract

Epidemics and pandemics of cholera, a severe diarrheal disease, have occurred since the early 19th century and waves of epidemic disease continue today. Cholera epidemics are caused by individual, genetically monomorphic lineages of *Vibrio cholerae*: the ongoing seventh pandemic, which has spread globally since 1961, is associated with lineage L2 of biotype El Tor. Previous genomic studies of the epidemiology of the seventh pandemic identified three successive sub-lineages within L2, designated waves 1 to 3, which spread globally from the Bay of Bengal on multiple occasions. However, these studies did not include samples from China, which also experienced multiple epidemics of cholera in recent decades. We sequenced the genomes of 71 strains isolated in China between 1961 and 2010, as well as eight from other sources, and compared them with 181 published genomes. The results indicated that outbreaks in China between 1960 and 1990 were associated with wave 1 whereas later outbreaks were associated with wave 2. However, the previously defined waves overlapped temporally, and are an inadequate representation of the shape of the global genealogy. We therefore suggest replacing them by a series of tightly delineated clades. Between 1960 and 1990 multiple such clades were imported into China, underwent further microevolution there and then spread to other countries. China was thus both a sink and source during the pandemic spread of *V*. *cholerae*, and needs to be included in reconstructions of the global patterns of spread of cholera.

## Author Summary

Cholera is a life-threatening, diarrheal disease caused by the bacterium *Vibrio cholerae*. After a long interregnum of decades without epidemics, the seventh cholera pandemic spread globally since 1961, causing considerable morbidity and mortality. Our analysis of published and newly sequenced genomes provides details on genetic groupings within *V*. *cholerae*, so-called clades, that have developed during the recent pandemic spread of these bacteria, and, in some cases, persisted to modern times. We reconstructed some of the pathways taken by the current pandemic since its origins in Indonesia, and show that both South Asia and East Asia are important pathogenic reservoirs and sources of international transmissions.

## Introduction

Cholera is an infectious and life-threatening diarrheal disease which is endemic in many African and Asian countries, and has also manifested as multiple, large epidemics and global pandemics since 1817 [1–3]. Older epidemics are attributed to the monophyletic ‘classical’ strains of *Vibrio cholerae* [4]. This attribution is supported by microbiological phenotypic typing which has been performed since the late 19^th^ century [2], and by the close genetic similarities between one genome from 1849 and those of several classical *V*. *cholerae* isolated in recent decades [4]. Between 1923 and 1959, classical *V*. *cholerae* remained endemic in India, and caused local cholera outbreaks in multiple countries, but pandemics did not occur. During that pandemic interregnum, a second phenotypic variant of *V*. *cholerae*, called ‘El Tor’, was also isolated from cholera patients, but only rarely. In 1961 a seventh cholera pandemic began and this has been predominantly associated with El Tor strains. Epidemiological records suggest that El Tor spread from the island of Sulawesi (formerly Celebes) in Indonesia to South and Southeast Asia, and then globally. Pandemic El Tor strains, including a sub-variant with an O139 surface polysaccharide, also corresponds to a monophyletic lineage, L2, which is closely related to other lineages from pandemic cholera, but clusters in a distinct phylogenetic branch [4–6]. During the seventh pandemic, successive sub-clusters of L2 genotypes are thought to have radiated in three waves from the Bay of Bengal [6] on the East coast of the Indian sub-continent, a region where cholera has been continuously endemic for centuries [2]. A large outbreak in Haiti in 2010 reflects the spread of wave 3 from South Asia [7], possibly from Bangladesh [8] or Nepal [9]. However, these reconstructions lacked information on the genetic composition of *V*. *cholerae* in China or eastern Asia, and were predominantly based on genomes from bacteria isolated in the 1970s, or thereafter.

**Fig 1:**
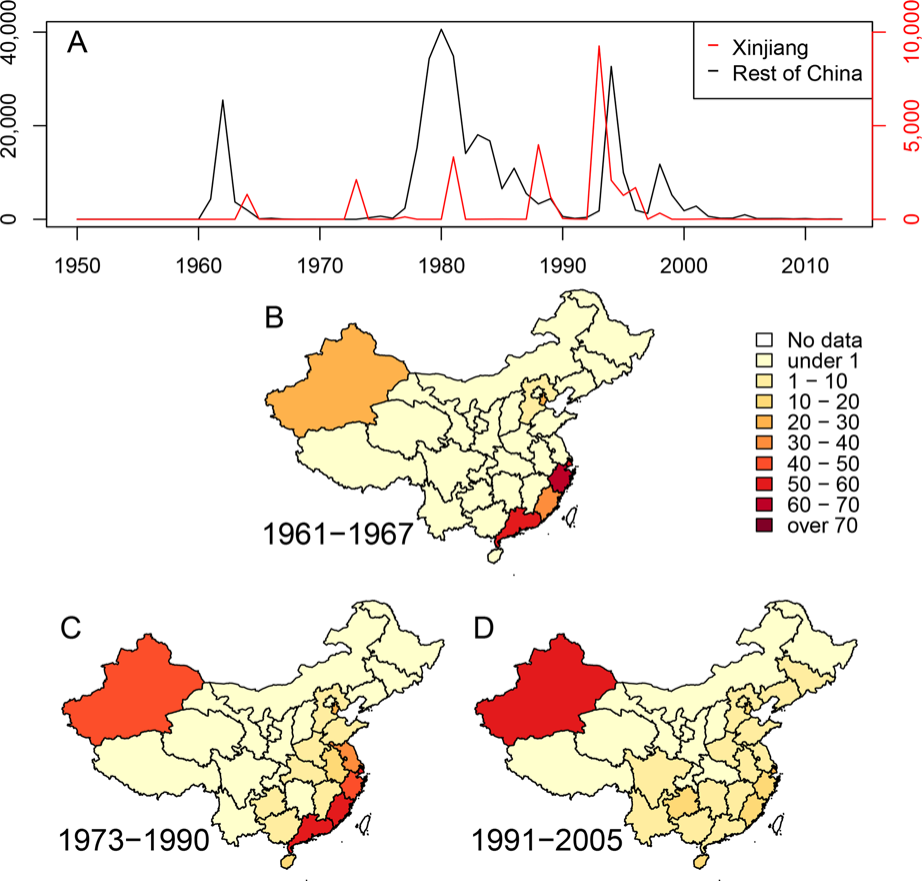
Cholera in China since 1950. (A) Numbers of cases of cholera per year in the Xinjiang region (red, scale at right) and the rest of China (black, scale at left). (B-D) Density of cases of cholera in China during three epidemiological waves of disease: 1961–1967, 1973–1990 and 1991–2005. Each province is colored by numbers of cases per million inhabitants per year.

It is clear from the epidemiological literature that cholera flared in China repeatedly between 1817 and 1923, following earlier outbreaks in South and Southeast Asia, and possibly spread from China to Japan, Korea, eastern Siberia and western Asia [2]. Outbreaks in China also broke out on multiple occasions between 1923 and 1959 [1]. A detailed reconstruction of the causes of these outbreaks, and their chains of transmission, is likely to be difficult because only very few bacterial isolates from those periods are known to exist. On the other hand, the period after 1961 is more readily amenable to analysis, and for integration into reconstructions of the spread of cholera in other parts of the world. Since 1961, three successive waves of cholera were recorded in Southeast and Central China [10], each involving many thousands of cases of disease caused by El Tor *V*. *cholerae* (Fig. 1). Multiple, partially overlapping outbreaks with fewer cases of cholera also occurred in in the Autonomous Region of Xinjiang in Northwest China. These observations might reflect successive flares of cholera from endemic sources of *V*. *cholerae* within China. Alternatively, China may have been a ‘sink’ for bacteria from external sources, and each wave in China might have resulted from an independent import of these bacteria from elsewhere. Under both hypotheses, the Chinese waves might additionally have acted as a ‘source’ for spread to neighboring countries and possibly even acted as an ‘amplifier’ of epidemic spread. In order to address these questions, we compared 260 genomes of *V*. *cholerae*, including 181 that had been previously analyzed [6,9,11], 71 newly sequenced genomes from strains isolated in China between 1961 and 2010, and eight from other sources (S1A Table).

## Results and Discussion

### Sources of the seventh pandemic

Mutreja *et al*. [6] concluded that all seventh pandemic isolates belonged to lineage L2, and estimated 1952 as the date for the most recent common ancestor (MRCA) of this lineage. Each L2 isolate differed from a reference seventh pandemic genome (N16961, Bangladesh, 1975) [12] by only 50–250 single nucleotide polymorphisms (SNPs). Other El Tor isolates were assigned to lineages L3, L5, L6 and L8 because they differed from N16961 by 3,000–6,000 SNPs. L3 and L8 contained recent El Tor strains from the US Gulf Coast and Australia, respectively. L5 contained two El Tor isolates from Sulawesi in 1937, prior to the seventh pandemic, and L6 corresponded to the oldest El Tor strain from 1910, which was isolated from an asymptomatic Indian pilgrim in El Tor, Saudi Arabia. However these analyses did not distinguish SNPs that were introduced by mutation, which can accumulate in a time-dependent, clock-like fashion, from clustered SNPs that are introduced by temporally unpredictable homologous recombination events involving DNA from distantly related bacteria, such as environmental *V*. *cholerae* [5]. Such distinctions are important because recombination can distort topological relationships, and artificially amplify genetic distances. The analyses of the history of lineage L2 by Mutreja *et al*. were based on strains which were isolated after 1975, with the exception of one isolate from Sulawesi (1957). Epidemiological records indicate that the seventh pandemic was preceded by small outbreaks in Sulawesi (1957) [13] and Ubol, northern Thailand (1959–60), and began with nearly simultaneous outbreaks in 1961 in multiple Indonesian islands, as well as in Malaysia, Macau, the Philippines and Hong Kong [14]. Starting in 1959, more than 60,000 individuals of Chinese extraction were resettled in southern China after expulsion from Indonesia because of their ancestry. They may also have brought cholera with them because a wave of cholera began in Southeast China in 1961–1963, followed in 1964 by outbreaks in Xinjiang (Fig. 1).

**Fig 2:**
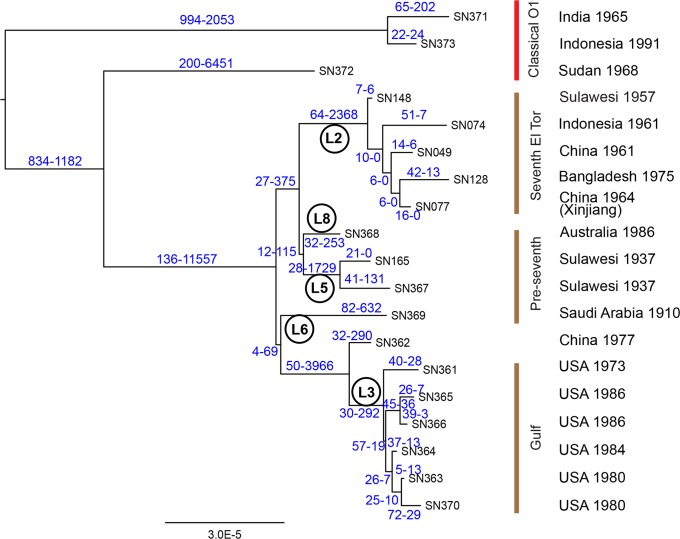
Phylogeny of 19 genomes from both El Tor and classical *V*. *cholerae*. The phylogeny is based exclusively on mutational SNPs (as identified using ClonalFrame), and the two numbers above each branch are the estimated numbers of SNPs caused by mutation and recombination, respectively. Lineage designations are indicated within circles. Strain designations (cf S1B Table) are indicated at the tips of the branches, and the source and date of isolation of each strain are shown at the right.

In order to clarify these issues, we re-examined the core genomic sequences of lineages L3, L5, L6 and L8, comparing them with genomes from classical strains as well as the earliest L2 strains isolated from Indonesia, China and Bangladesh. We used ClonalFrame [15] to estimate for each SNP the probability of having arisen by mutation or recombination, and calculated a maximum likelihood tree based exclusively on mutational SNPs (Fig. 2). Interestingly, only 336 mutational SNPs separated the root of all El Tor lineages from the most closely related classical genome (SN372). Furthermore, the MRCA of L2 only differed by 76 mutational SNPs from the MRCA of L5 (Sulawesi, 1937) and L8 (Australia, 1986), suggesting that their common ancestor existed quite recently. That ancestor differed by 31 mutational SNPs from the MRCA of L6 (Saudi Arabia, 1910), L3 (Gulf strains) and a closely related Chinese isolate from 1977. The L2 lineage encompassed not only the early seventh pandemic isolates but also the slightly earlier isolate from Sulawesi (1957). Individual L2 genomes differed from each other by 36–105 mutational SNPs, which is similar to the pairwise differences between El Tor lineages.

The individual lineages within classical and El Tor genomes were previously distinguished because they defined long branches [4–6]. Our analysis indicates that the length of those branches was largely due to recombination, which introduced thousands of clustered SNPs (Figs. 2, S1), including 2,368 SNPs on the branch leading to the MRCA of lineage L2. However, very little of the diversity between the five L2 genomes was attributed to recombination (32 SNPs). The results in Fig. 2 also provide an initial perspective on the evolutionary genealogy that led to the seventh pandemic. At the base of L2 are two isolates from Indonesia sampled in 1957 and 1961, suggesting that this is the true source of the seventh pandemic. A close relationship was found between an isolate from the Chinese province of Xinjiang (1964) and one from Bangladesh (1975). Outbreaks of cholera caused by El Tor strains were first reported in Bangladesh, India and Pakistan in 1963, 1964 and 1965, respectively [16]. These countries border on Xinjiang or are not very distant (S2 Fig), and cross-border exchanges are frequent enough between the Muslim populations in these areas that in 2011, an epidemic of poliomyelitis in Xinjiang was imported from Pakistan [17]. *V*. *cholerae* could also have been transmitted across these country borders in the early 1960’s but no genomes are yet available from that period except for the Chinese isolates described here.

### Hypermutators in the genealogy of lineage L2

**Fig 3:**
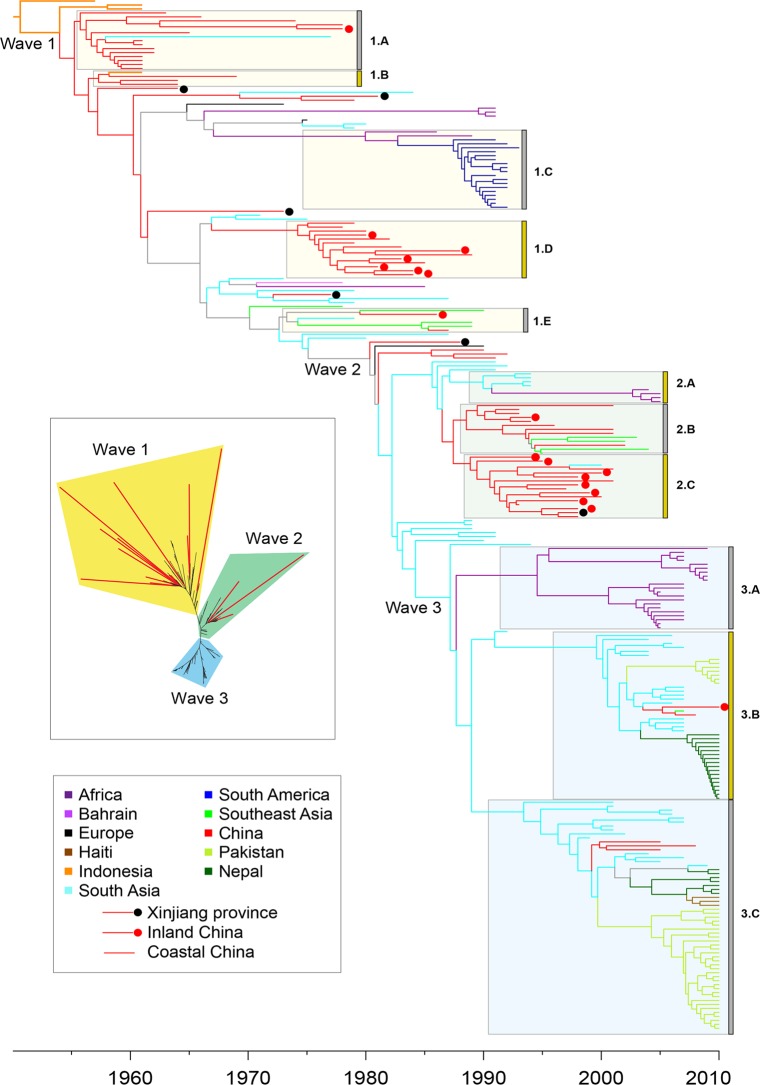
Timed phylogeny for 6,335 SNPs in 260 genomes from the seventh pandemic. The vertical order is the same as in S1A Table. Branches are colored according to inferred location as shown in the legend at the lower left, with the exception of branches for which the location was uncertain which are shown in gray. Isolates from China are subdivided into isolates from Xinjiang (black dot), inland provinces (red dot) and coastal provinces (no dot). Selected clades of multiple, closely related isolates are indicated by grey boxes next on the left of the clade designations (1.A, 1.B, etc). Inset: Maximum likelihood tree of the same data with significantly longer branches according to S3 Fig indicated in red.

We now focus on the seventh pandemic based on 260 genomes from lineage L2. A maximum likelihood tree based on the 6,335 SNPs in their non-repetitive, core genomes (Fig. 3 inset; S3A Fig) confirmed that they are all genetically related, and clustered in three successive groups of decreasing diversity, which correspond to the three waves that were previously described [6]. The root-to-tip distances in the phylogeny correlated strongly with the dates of isolation of the individual strains (S3B Fig; R^2^ = 0.932; p-value = 2.56x10^-142^) with the exception of 17 of the 260 genomes which were significant outliers to this linear regression. They were located on long terminal branches (red in Fig. 3 inset and S3 Fig), as previously noted for strain A4 [6].

Several possibilities could account for these long branches. They could represent sequencing mistakes, but this was ruled out because manual re-sequencing confirmed all 44 randomly selected SNPs from long branches that were tested. Long branches can also result from recombination, but here we found none of its typical signatures. Firstly, only 2% (125) of the SNPs were homoplastic, that is unexplainable by a single mutation on the tree. Secondly, the relative effect of recombination to mutation (r/m) was equal to 0.1 according to a ClonalFrame analysis, with only ~1% of the core genome affected by recombination on any branch in the phylogeny. Thirdly, the SNPs on the long branches were spread evenly around the genome (S4 Fig), rather than clustered as would be expected from recombination [18], and as found in our comparisons between lineages (S1 Fig). These observations confirm that recombination has been very rare in L2, and exclude it as a likely cause of the long branches.

Long branches can also be caused by the elevated mutation rates in hypermutators, which arise naturally in populations of *Escherichia coli* [19–21], *Neisseria meningitidis* [22] and *Yersinia pestis* [23] following disruption of the mismatch repair system. Hypermutators may promote the acquisition of antibiotic resistance [24] or other forms of adaptive evolution [25], but in the longer term their high mutation rate results in reduced fitness [26] and they do not succeed in establishing themselves against the competition of non-mutators. To test this possibility, we measured the *in vitro* mutation rate of all 79 strains at our disposal, including 16 on long branches (S2 Table). The strains associated with long branches had significantly higher mutation rates than the others (S5 Fig, Kruskal-Wallis test, *p*-value = 8.44x10^-4^). Most of them were also associated with mutations that can lead to the hypermutator phenotype: 14 of the 17 genomes on long branches possessed a total of 18 genetic variations in one or more of four genes (*mutS*, *mutH*, *mutL* and *uvrD*) that play a key role in the mismatch repair system (S3 Table). These 18 variations included ten short indels resulting in frameshifts, five non-synonymous codon changes and one premature stop codon for a total of 16 changes in protein sequence versus two synonymous mutations. In contrast, only four of the 243 genomes with normal branch lengths had changes in the amino acid composition encoded by one of these four genes (Fisher exact test, p = 6x10^-17^). In ten of the genomes with long branches, we also identified 12 non-synonymous changes, one frameshift, one premature stop codon and two synonymous mutations in ten other genes that can affect mismatch repair (S3 Table).

The majority (9/17) of the strains with long branches were isolated between 1961 and 1965, relatively soon after the beginning of the seventh pandemic. It is tempting to speculate that a high frequency of hypermutators was causally associated with the rapid spread of the seventh pandemic, especially because hypermutators may be a sign of recent selective pressure and population bottlenecks. However, these old strains of *V*. *cholerae* had been maintained in stab cultures for many years, which also tends to select for mutations [27]. Thus, confirming the importance of multiple hypermutators among early strains from the seventh pandemic will require the analysis of additional old strains that have not been stored as stab cultures.

### Long-term chains of transmission

We estimated a timed phylogeny for the SNPs among the 260 genomes in which leaves are aligned with their isolation dates, and branch lengths represent time rather than number of mutations. Due to the presence of the hypermutators, the phylogeny was calculated using a relaxed molecular clock model [28] in which the 17 long terminal branches each had their own independent evolutionary rate (Fig. 3). The evolutionary rate for the remainder of the tree was estimated as ~2.3 substitutions per genome per year, and the MRCA of the seventh pandemic was dated to 1954. These results are in good agreement with Mutreja *et al*. [6], and similar to the clock rates estimated for multiple other bacterial pathogens [29]. According to the timed phylogeny, the three previously described waves do not seem well defined: they simply correspond to three internal branches with multiple descendants (Fig. 3). Furthermore, the three wave concept oversimplifies the epidemiological patterns, because the timed phylogeny does not correspond to successive, discrete radial expansions from single nodes. Instead, each of the waves is preceded by multiple, closely related long branches which currently contain only few isolates. As additional historical isolates are examined, it may become even more difficult to determine the exact position within the tree where initiated each of these supposed waves.

A second problem also exists with the wave concept, namely that epidemiological inferences have been used to designate phylogenetic structures, which implies causality that may not exist. We therefore recommend substituting neutral designations for well-defined monophyletic clades where multiple genomes cluster tightly, as a replacement for wave designations. Such designations would facilitate testing for an association between phylogenetic clade, genomic content and increased transmission. We have therefore assigned designations (1.A, 2.B, etc.) to several obvious monophyletic clades, including the prior wave number in order to support comparisons with prior publications as well as arbitrary letters (Fig. 3; S1A Table). All but three of 123 strains in wave 3 from global sources were isolated after 2000, and cluster in clades 3.A, 3.B and 3.C. Clade 3.A corresponds to an African epidemic which seems to have been imported from South Asia. Clades 3.B contains strains from the outbreak in Haiti, as well as from South Asia (Nepal, Bangladesh) and China (two strains). Clade 3.C contains strains from South Asia and Pakistan as well as multiple, other global sources, including three from China. These patterns are consistent with China having been a sink for *V*. *cholerae* since 2000, rather than a source.

Most of the strains isolated in China between 1991 and 2005 belonged to clades 2.B (Coastal China) and 2.C (Inland China). Clade 2.B was ancestral to several genomes from Southeast Asia; and was preceded in the phylogeny by still other genomes from South Asia, but progenitors of these clades were also isolated in China, including an isolate from the Xinjiang province in 1988. These observations are consistent with China having been an initial source for the global spread of these clades and their relatives, but do not preclude later strains having been imported from outside. China also seems to have played a role in the earliest global transmission of the 7^th^ pandemic after its origins in Indonesia. Apart from the three earliest strains from Indonesia, the deepest branches (clades 1.A and 1.B) were found in China in the 1960s. Clade 1.C contained multiple isolates from South America in the 1990s, and is derived from the deeper branches in clade 1.B that were found in China. Chinese isolates are found on all subsequent deep branches (for example in clade 1.E), as well as forming several terminal branches but most Chinese isolates from the 1973–1990 outbreak cluster in clade 1.D. Several intermediate branches in this early phase of spread were from Xinjiang, providing further support for transmission to other countries from Northwest China.

**Fig 4:**
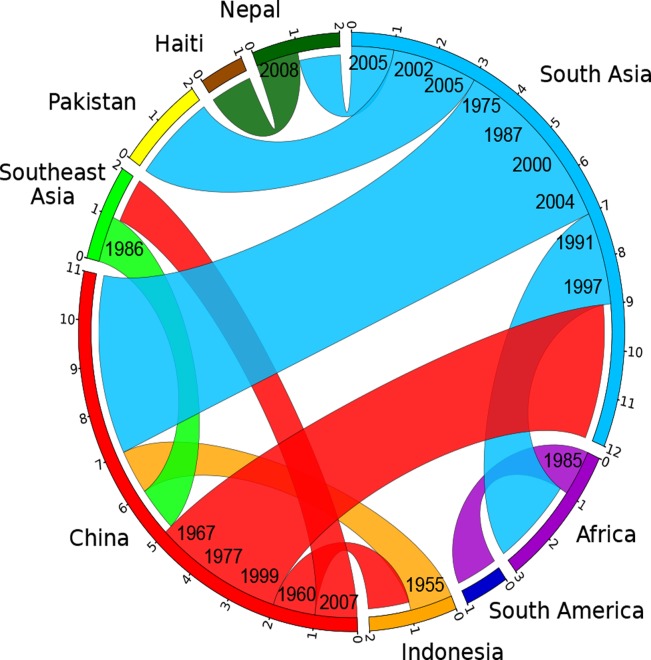
Circular plot illustrating the inferred migrations between geographical locations. Flow bars indicate the sources of transmissions, colored as in Fig. 3, with one end of the bar directly touching the country of origin, and the other end of the bar having a small gap before the country of destination. The average date for each migration is shown on the ends corresponding to the origin.

In order to investigate these source/sink relationships in greater detail we inferred the ancestral geographical locations of branches by a maximum parsimony reconstruction of states, summarizing sources and sinks for international transmissions by a circular plot [30,31] (S4 Table; Fig. 4). Our data indicate nine transmissions out of South Asia, including twice into Africa in the 1990s, twice into Pakistan between 2002 and 2005, and once into Nepal around 2005. The strain causing the Haitian 2010 outbreak was confirmed to have originated in Nepal, as previously suggested [9]. Likewise, we also confirmed pandemic spread from Africa into South America in the 1980s [6]. These conclusions should be considered as minimal estimates of the numbers of transmissions because numerous sources of international outbreaks and endemic disease have not been investigated, leading to sampling bias. In contrast, more genomes have now been sequenced from China than from any other single source, and the Chinese strains are representative of disease over the entire period from 1961 until 2005. Our analysis of global transmissions (Fig. 4) indicates that China imported *V*. *cholerae* four times from South Asia (1975–2004), once from Indonesia (1955) and once from Southeast Asia (1986). In turn, China was the source of transmissions to South Asia (three times, 1967–1999), Indonesia (1960) and Southeast Asia (2007). These results suggest that *V*. *cholerae* populations are often transmitted between East Asia, South Asia and Southeast Asia, which makes it difficult to pinpoint the exact origins or outbreaks in other parts of the world. Even if China is not the direct origin for such outbreaks, it clearly represents an important reservoir of diversity which needs to be accounted for when modeling the global epidemiology of cholera.

## Materials and Methods

### Isolates

Metadata for the 260 lineage L2 strains whose genomes were compared is presented in S1A Table, including dates and countries of isolation and accession numbers. 79 of these isolates were newly sequenced for this study from a previously described collection [10], of which 71 were from China. The analysis also included previously sequenced genomes from 119 isolates from a global collection [6], 24 from Nepal [9] and 38 from Pakistan [11], and the tree was rooted with the pre-seventh pandemic strain M66 [32], which belongs to lineage L5 (cf SN165 in Fig. 2). Five of these 260 genomes from L2 plus 14 genomes from other lineages than L2 were used in Fig. 2, as listed in the S1B Table.

### Experimental mutation rate

For each of the 79 newly sequenced isolates, the mutation rate to rifampicin resistance was experimentally measured as previously described [33]. Briefly, 10^2^ to 10^3^ cells from an overnight culture were inoculated on nitrocellulose filters which had been placed on 869 plates. After incubation at 37°C for 24 h, the cells were re-suspended in 1 ml of 869 broth and incubated at 37°C for 1 h to allow expression of rifampicin resistance. Appropriate dilutions were then spread in parallel on both 869 plates and 869 plates containing 100ìg/ml of rifampicin. The rifampicin resistant mutants were counted after incubation at 37°C for 24 h, and each mutation rate was calculated as the median of six independent cultures (S2 Table).

### Genome sequencing

DNA was prepared from 1 ml overnight cultures with the Wizard Genomic DNA Purification Kit (Promega). Whole genome sequencing was performed at BGI (China) on 78 genomes using an Illumina HiSeq 2000 on 250 bp and 6 kb paired-end libraries in 100-fold multiplexes (see S1 Table for number of reads, read length and N50 statistics for each genome), whereas the finished genome of strain FJ147 was obtained on an ABI Prism 3730 with Sanger sequencing (BGI, China).

### Assembly and identification of SNPs

We applied two independent methods to assemble contigs and call SNPs for the 260 genomes plus outgroup strain M66. First we performed reference-based mapping against the reference genome of N16961 [12] using Bowtie 2 [34]. Secondly, we performed *de novo* assembly using SPAdes [35] followed by SNP calling against N16961 with MuMMER [36]. The two methods identified 9,064 and 9,089 SNPs respectively, of which 8,987 SNPs were identical between both methods and were therefore used for further analysis. 2,652 of these SNPs were specific to the outgroup strain M66, leaving 6,335 SNPs differentiating the 260 genomes from the current pandemic.

### Confirmation of SNPs in genomes on long terminal branches

We randomly selected 44 SNPs from 16 long terminal branches in S3 Fig. For each SNP, the flanking 250bp on both sides from the assembled genome were used to design amplification and sequencing primers with Primer3 [37], which were then used for Sanger sequencing of the genomic SNP.

### Phylogenomic analysis

In order to compare the 19 genomes (listed in S1B Table) from both Classical and El Tor *V*. *cholerae*, ClonalFrame [15] was first run to determine the sites likely to have been affected by recombination with posterior probability above 50%. A maximum-likelihood tree (Fig. 2) was then constructed based on the non-recombinant sites using PhyML 3.0.1 [38]. For each branch of the phylogeny, the expected numbers of SNPs caused by mutation and recombination were estimated using ClonalFrame.

In order to compare the 260 genomes (listed in S1A Table) from the seventh pandemic, a maximum-likelihood tree (Fig. 3 inset; S3A Fig) was computed by applying PhyML 3.0.1 [38] to the 8,987 SNPs differentiating the genomes between themselves and from the pre-seventh pandemic genome M66 which was included as an outgroup to root the tree. The significance of abnormally long branches in this phylogeny was tested using a strict clock molecular clock rate model, which identified 17 branches that were significantly longer than expected (red branches in Fig. 3 inset and S3A Fig; red dots in S3B Fig). We therefore calculated a timed phylogeny (Fig. 3) using a previously described method [39], which consists of finding the posterior distribution of the dates of the ancestral nodes and rates of the clock model given the observed number of substitutions on each branch. This inference was performed under the assumption of a local molecular clock model [28] with a total of 18 parameters to allow a separate clock rate for each of the 17 long branches and a single rate for the remainder of the tree. The geographical location of terminal branches was the known place of isolation of the strains. For internal branches, the likely location was reconstructed using maximum parsimony ancestral reconstruction [40], which identified a minimum of 37 migration events to explain the data. Of these, 18 were unambiguous (meaning that they could only have a single source and a single destination) and correspond to a change of color from parental to daughter branch in Fig. 3, with the exception of grey branches where multiple ancestral locations were probable. The sources and destinations of the 18 unambiguous migrations are summarized in S4 Table, along with the estimated date of each migration which was calculated as the date at the middle of each migrant branch. This data was also represented as a circular plot (Fig. 4) using the Circos [30] table viewer (available at http://mkweb.bcgsc.ca/tableviewer/), to represent tabular data in a graphical circular format.

### Data deposition

The sequence data have been deposited with the European Nucleotide Archive, www.ebi.ac.uk/ena accession number ERP006431–50 (PRJEB6790–809), ERP006452–5 (PRJEB6811–814) and ERP006457–510 (PRJEB6816–869). The complete genome sequence of FJ147 was deposited to GenBank under accession number CP009041–2. All accession numbers are listed in S1 Table.

## Supporting Information

S1 Fig Output of ClonalFrame based on 19 genomes of both classical and El Tor *V*. *cholerae*. The clonal genealogy reconstructed by ClonalFrame is shown on the left. For each branch of this tree there is a row in the heat map on the right, which shows the probability of recombination estimated by ClonalFrame along the genome. These probabilities are color-coded from 0 to 1 according to the legend shown at the top.
(PDF)

S2 Fig Geographical map of the Autonomous Region of Xinjiang in China and neighboring countries. (PDF)

S3 Fig (A) Maximum likelihood phylogeny for 260 genomes of *V*. *cholerae* from the current pandemic. Labels are not shown, but the vertical order is the same as in S1A Table, and every 10^th^ branch is marked by a dot. This is the same phylogeny as shown in Fig. 3 inset. (B) Scatter plot of the relationship between isolation date on the X-axis and the root-to-tip distance in the phylogeny shown in part A for all 260 genomes. The 17 isolates that fall out of the expected distribution are marked in red in both parts A and B.
(PDF)

S4 Fig Genome-wide distribution of SNPs that are unique to the 17 exceptional genomes with long terminal branches. A red dotted line separates the two chromosomes.
(PDF)

S5 Fig Histograms showing the experimentally measured mutation rates for 63 strains with typical branch lengths (top) *versus* 16 strains with exceptionally long branches (bottom). (PDF)

S1 Table List of genomes used in this study. Part A: list of 260 genomes used in Fig. 3, in the same order from top to bottom. Part B: list of 19 genomes used in Fig. 2, in the same order from top to bottom.
(XLSX)

S2 Table Mutation rates measured experimentally for 79 strains. (XLSX)

S3 Table Variations in genes of the mismatch repair system among 17 genomes with long branches. (XLSX)

S4 Table Summary of the 18 unambiguous international migration events reconstructed by maximum parsimony, including their source (row), destination (column) and estimated date (in brackets). (XLSX)
