# Supplementary figures and images for: The Role of China in the Global Spread of the Current Cholera Pandemic
Source: PLoS Genet. 2015 Mar 13;11(3):e1005072. doi: 10.1371/journal.pgen.1005072 (PMC4358972; doi:10.1371/journal.pgen.1005072)

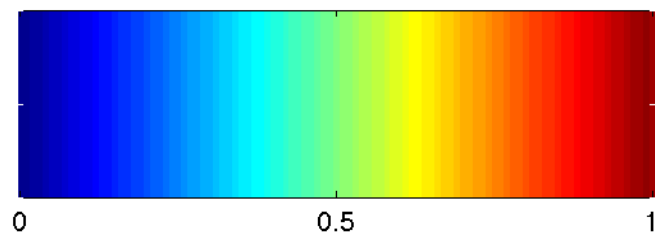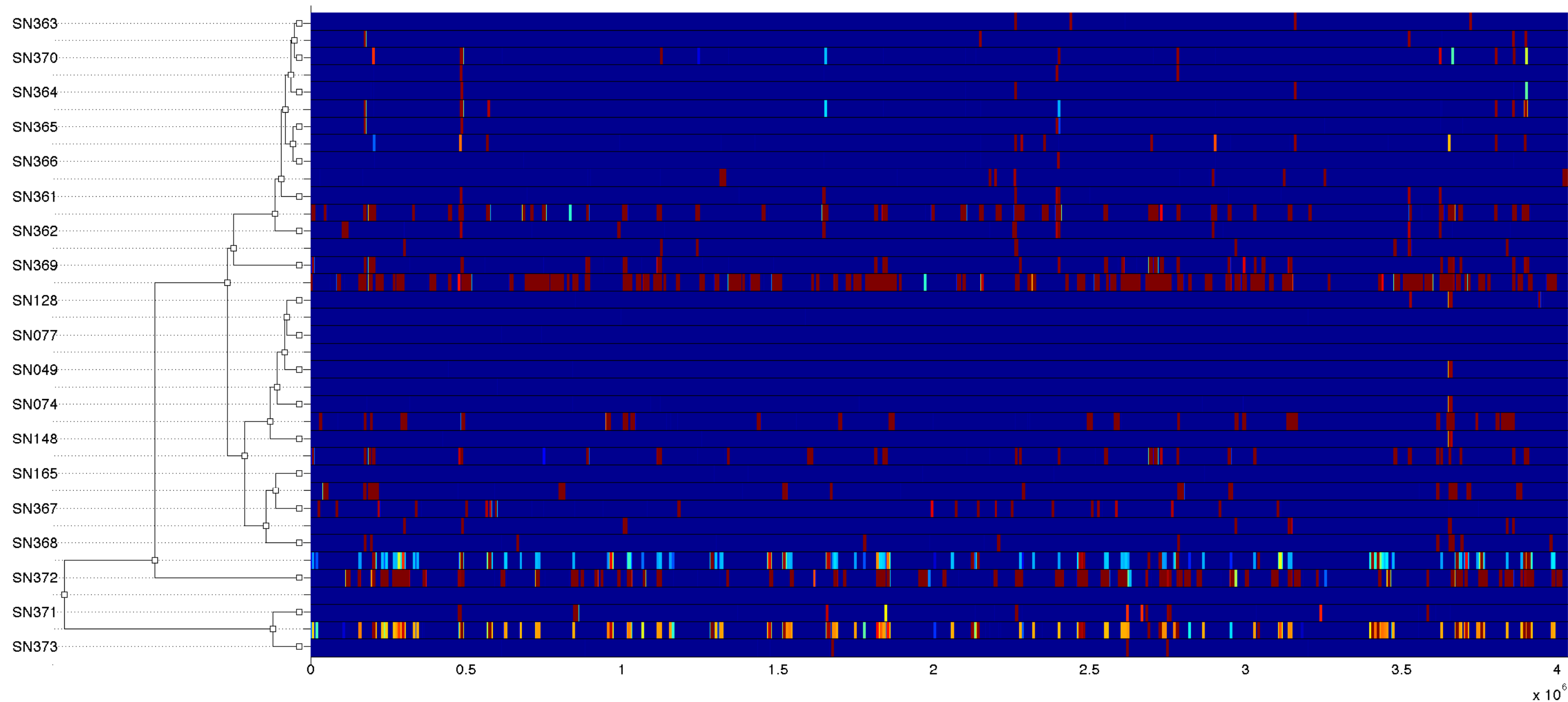

Supplement: S1 Fig — The clonal genealogy reconstructed by ClonalFrame is shown on the left. For each branch of this tree there is a row in the heat map on the right, which shows the probability of recombination estimated by ClonalFrame along the genome. These probabilities are color-coded from 0 to 1 according to the legend shown at the top. (PDF) [file pgen.1005072.s001.pdf]

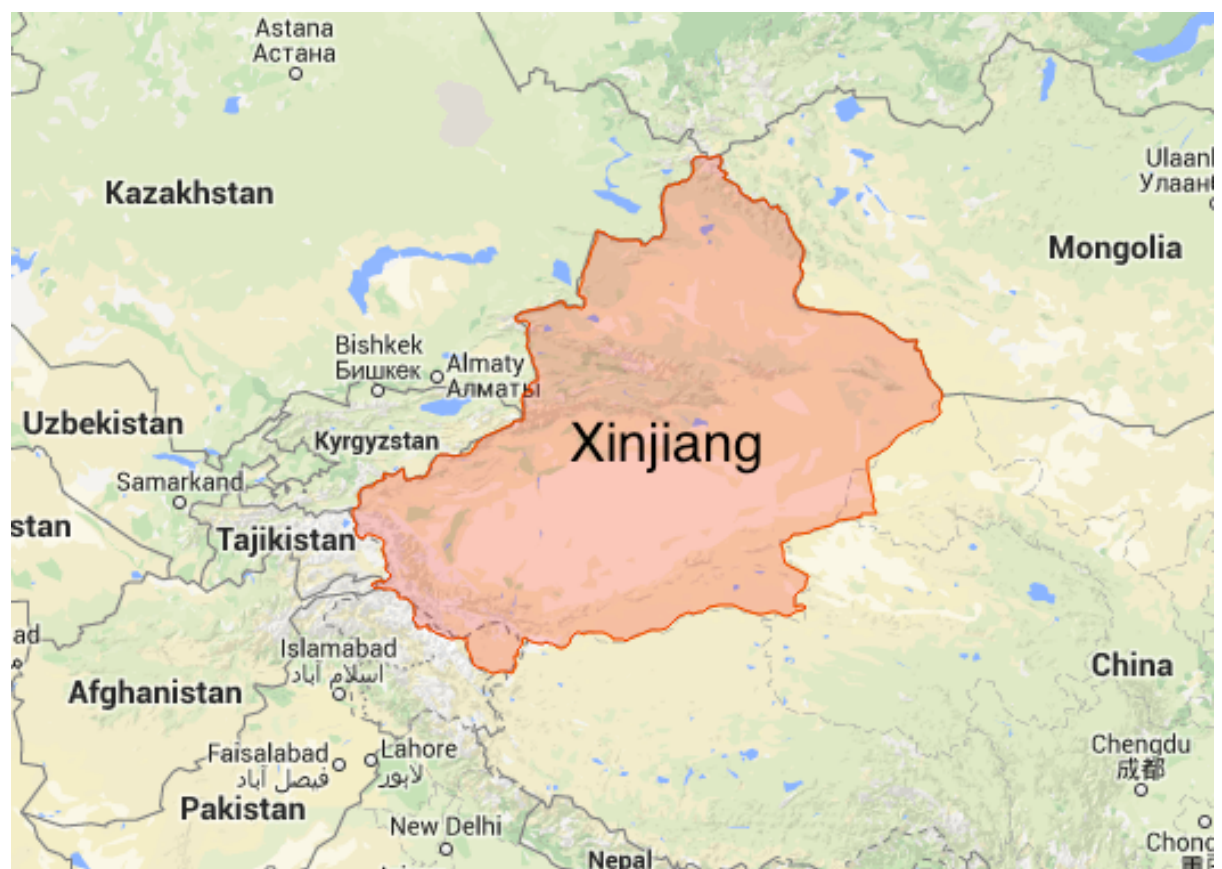

Supplement: S2 Fig — (PDF) [file pgen.1005072.s002.pdf]

**A**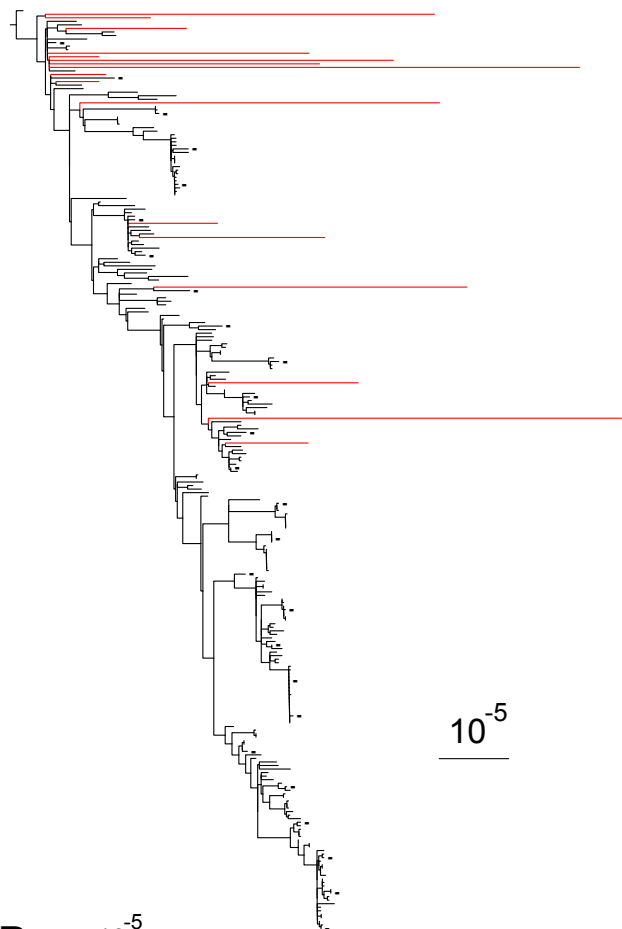**B**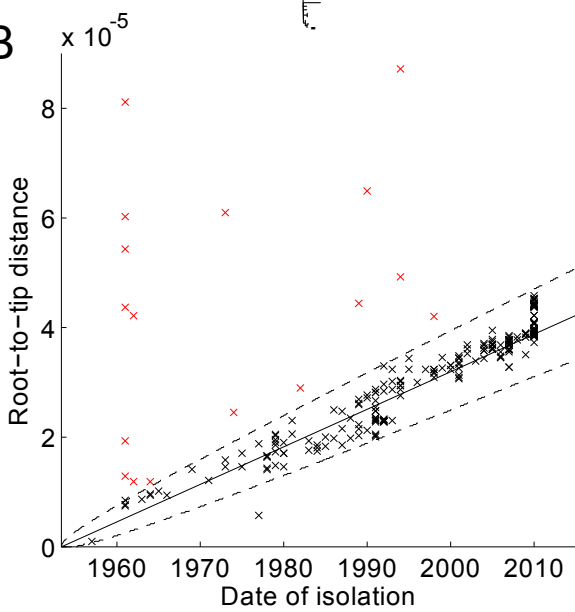

Supplement: S3 Fig — Labels are not shown, but the vertical order is the same as in S1A Table, and every 10th branch is marked by a dot. This is the same phylogeny as shown in Fig. 3 inset. (B) Scatter plot of the relationship between isolation date on the X-axis and the root-to-tip distance in the phylogeny shown in part A for all 260 genomes. The 17 isolates that fall out of the expected distribution are marked in red in both parts A and B. (PDF) [file pgen.1005072.s003.pdf]

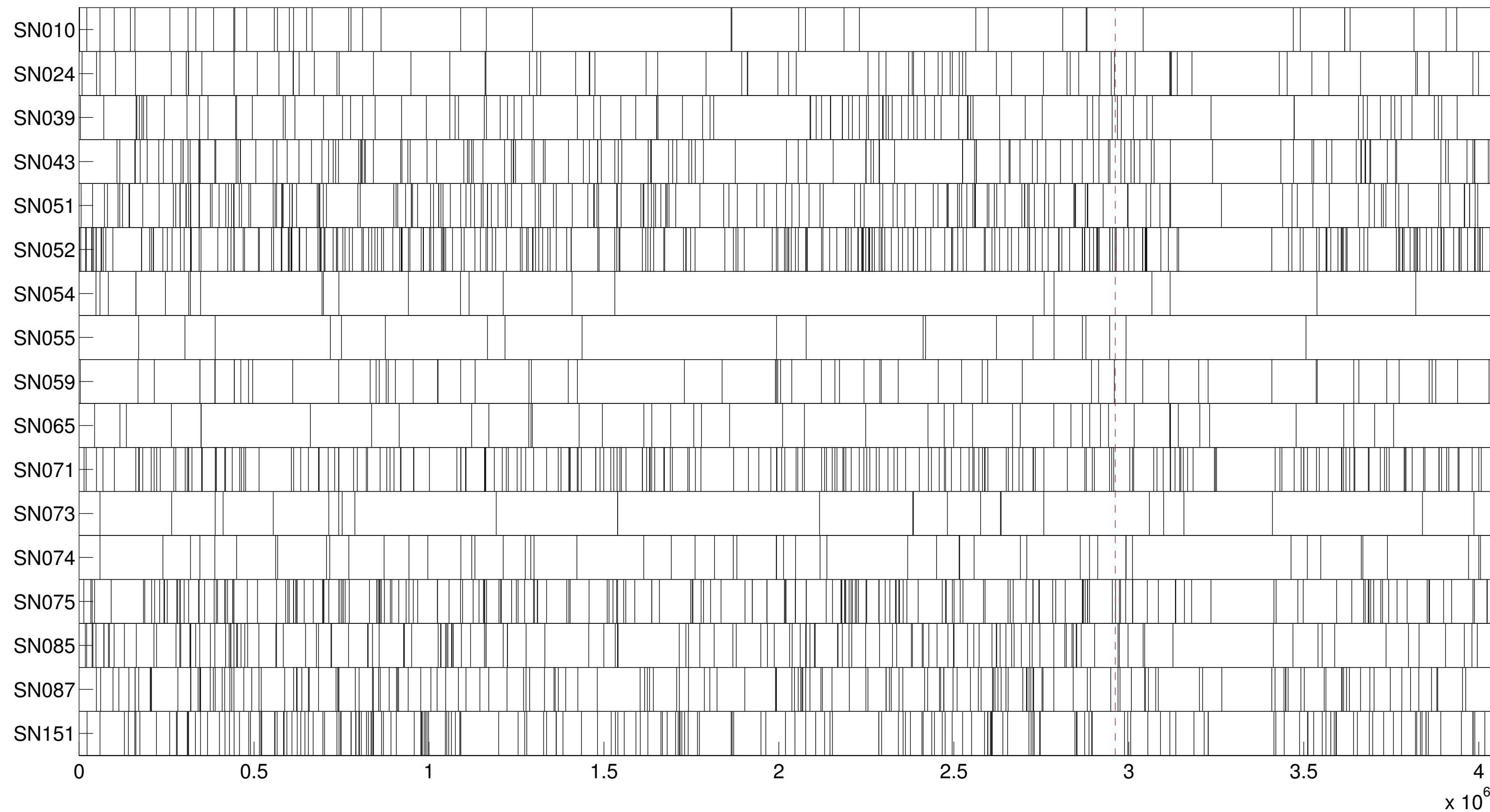

Supplement: S4 Fig — A red dotted line separates the two chromosomes. (PDF) [file pgen.1005072.s004.pdf]

Branches of normal length

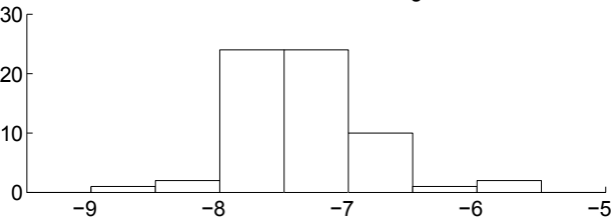

Branches longer than expected

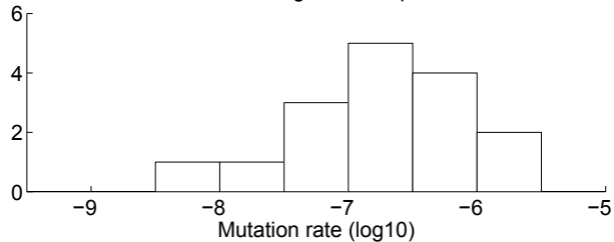

Supplement: S5 Fig — (PDF) [file pgen.1005072.s005.pdf]
